# Georeferenced data employed in the spatial analysis of neighborhood diversity and creative class share in Chicago

**DOI:** 10.1016/j.dib.2015.07.008

**Published:** 2015-07-16

**Authors:** Bradley Bereitschaft, Rex Cammack

**Affiliations:** University of Nebraska at Omaha, United States

## Abstract

The dataset described in this article, and made available as an accompanying spreadsheet, was used in the study entitled, “Neighborhood diversity and the creative class in Chicago,” to assess the spatial associations between neighborhood diversity and the creative class at the neighborhood (i.e., census tract) scale in Chicago [1]. In this study, we found a significant positive association between the creative class and the proportion of gay households and income diversity, but not racial or linguistic diversity. However, a geographically-weighted regression (GWR) analysis demonstrated substantial spatial nonstationarity among these relationships. This article describes the creative class, diversity, and control variables, their sources, and the methods used to calculate them.

*Correspondence to: Department of Geography/Geology, University of Nebraska at Omaha, 6001 Dodge Street, Durham Science Center 263, Omaha, NE 68182, USA.

**Table:** 

Subject area	Geography
More specific subject area	Urban geography.
Type of data	Excel spreadsheet containing all creative class, diversity, and control variables for each census tract in the Chicago study area.
How data was acquired	Occupational and demographic data were obtained from the U.S. Census Bureau; land cover data from the USGS; grade school rankings from the Chicago Sun Times; and transit data from the Chicago Regional Transportation Authority. Diversity variables were calculated using Simpson׳s index of diversity.
Data format	Raw and processed.
Experimental factors	
Experimental features	
Data source location	
Data accessibility	The data is with this article.

**Value of the data**

• Where the “creative class” [4] prefers to live within cities, and why, may have important implications for spatial inequality and segregation.
• This dataset provides an exploration of some of the factors that might attract and “anchor” the creative class at the intra-urban scale.
• Census tract-level data on neighborhood diversity, access to urban amenities, and the proportion of workers employed in specific creative class occupations in Chicago may be used in a wide variety of urban research.

## 1 Study area

Data were georeferenced to 1983 census tracts within the city of Chicago and seven surrounding counties in northwestern Illinois: Cook (of which the City of Chicago is the county seat), Lake, McHenry, Kane, DuPage, Kendall, and Will. Data not already aggregated at the census tract level (e.g., proximity to rail stations, top schools) were georeferenced to the geographic center, or centroid, of the census tract.

## 2 Data collection and processing

The complete dataset consists of four measures of diversity, the proportion of workers employed in five separate creative class occupational groupings, and seven urban amenity variables used as controls. Demographic, socio-economic, and occupational data used to estimate diversity and creative class occupational share by census tract were obtained from the U.S. Census Bureau (U.S. Census Bureau 2012). All census data are five-year averages (2008–2012) Table 1.

### 2.1 Creative class variables

Richard Florida [4,5] identified two major groups of creative class workers: the super creative core and creative professionals. Creative professional occupations include management, business, finance, legal, health care and high-end sales. The super creative core consists of workers with the most creativity-intensive occupations, including computer/math, architecture/engineering, life/physical/social science, education/training/library, and arts/design/entertainment/sports/media. Our analysis utilized five separate creative class groupings based on the U.S. Census 2010 classification scheme: the proportion of workers with any creative class occupation, the proportion of workers with any super creative core occupation, and those with more specific super creative core occupations including computer/engineering/science (CES), education/library/training (ELT), and arts/design/entertainment (ADE). The proportion of workers with creative class occupations were calculated for each census tract using all civilian workers aged 16 years or older.

### 2.2 Neighborhood diversity variables

Diversity variables included sexual orientation (i.e., the percent of gay households in each census tract), race, dominant language spoken at home, and median household income. Racial, linguistic, and income diversity for each census tract were calculated using the Simpson׳s Index of Diversity

(1) $$y=1-\sum_{k}{\left(\frac{n}{N}\right)}^{2}$$

where *n* is the number of residents of a particular category, and *N* is the total number of residents per census tract [9]. The index varies from 0 to 1, with higher values indicative of higher diversity. The index measures the likelihood that two individuals selected at random will belong to separate racial/linguistic/income categories. Race was divided into seven census-defined categories: White, Black, Native American, Asian, Pacific Islander, Hispanic, and ‘some other race.’ Linguistic diversity was estimated using eight categories based on the seven most common languages spoken at home within the Chicago study area (i.e., English, Spanish, Polish, Chinese, Tagalog, Korean, and German) plus an additional category to represent all other languages. The income diversity index was based on four consolidated census income categories representing low income ($0–24.9*k*), low middle income ($25–59.9*k*), high middle income ($60–99.9*k*), and high income (100*k*+) households. The four income categories were chosen to align as close as possible with the study area׳s household income quartiles.

In addition to assessing and reporting diversity for each individual census tract separately, a geoprocessing model developed in the ArcGIS™ v. 10.2 ModelBuilder was used to compute a neighborhood average for the diversity indices and the percentage of gay households at each census tract. The geoprocessing model used an iteration approach combined with a spatial query and statistical method. All census tracts in the study area were iterated over, and a spatial query was performed using spatial touching logic [2]. For each census tract, all adjacent tracks including those touching only at the corner (i.e., “Queens case” contiguity; [3]) were selected and an average for each of the variables was calculated. The model then repeated the procedure with a new tract to reinitiate the spatial query. The spatial averages were added to the list of attributes for each tract as the model progressed. Both the individual census tract value and neighborhood value are reported in this dataset. For the smaller and more densely populated urban census tracts in particular the averaged measure may provide a more accurate representation of diversity at the neighborhood scale. Furthermore, the incorporation of alternative spatial units in geographic analyses is a common practice due to the Modifiable Areal Unit Problem (MAUP). The MAUP is the tendency for data sets to change when using different geographic units of analysis [10].

### 2.3 Control variables

In the process of assessing the relationships between the proportion of workers with creative class occupations and neighborhood diversity, it was necessary to include in the modeling procedure a number of control variables. The seven control variables aggregated at the census tract level include: (1) land value (as approximated using median home values), (2) proximity to ‘top’ grade schools and (3) colleges/universities, (4) presence of water and open space, (5) proximity to rail stations and (6) ‘third places’, and (7) population density. ‘Top’ grade schools included 122 elementary, middle, and high schools within the Chicago study area. Schools were ranked by the *Chicago Sun Times* [8] using standardized Illinois state achievement exam scores. Distance from the center of each census tract to the closest ‘top’ school was used to estimate proximity. The same methodology was used to calculate proximity to colleges/universities and rail stations. Colleges and universities included all non-profit institutions of higher learning within the Chicago study area, totaling 160 separate campuses. Both Meta (Chicago׳s commuter rail network) and ‘L’ (rapid transit) stations were used in the calculation of rail station proximity. Proximity to rail was calculated as the distance to the nearest rail station, whether Meta or L. In addition to the overall proximity measure, the minimum distance to both rail stations are reported for each census tract. Using the Multi-Resolution Land Characteristics Consortium׳s 2011 National Land Cover Database [6], the amount of land use classified as open space or water within a two kilometer radius of each census tract centroid approximated the availability of recreational and scenic amenities. Lastly, proximity to ‘third places’, was calculated by averaging the distance from each census tract centroid to the five nearest establishments. ‘Third places’ are [typically consumption] spaces separate from home (the ‘first place’) and work (the ‘second place’) that facilitate casual social interactions [7]. ‘Third places’ included coffee shops, bars, pubs, lounges, bookstores, and deli-bakeries. The location and attributes of ‘third places’ were identified using the ReferenceUSA^®^ online database.

## Figures and Tables

**Table 1:** List of variables included in this dataset.

Diversity variables	Creative class variables	Control variables (amenities)
% Gay households	Creative class (total)	Median home value
Racial diversity	Super creative core	Proximity to ‘top’ grade schools
Linguistic diversity	Computer, Engineering, Science	Proximity to college/university
Income diversity	Education, Library, Training	Presence of open space
	Art, Design, Entertainment	Proximity to rail stations
		Proximity to ‘third places’
		Population density
